# Anatomy of Teaching Anatomy: Do Prosected Cross Sections Improve Students Understanding of Spatial and Radiological Anatomy?

**DOI:** 10.1155/2016/8984704

**Published:** 2016-08-08

**Authors:** L. B. Samarakoon, S. Vithoosan, S. Kokulan, M. M. Dissanayake, D. J. Anthony, Vajira Dissanayake, Rohan Jayasekara

**Affiliations:** Department of Anatomy, Faculty of Medicine, University of Colombo, No. 25, Kynsey Place, 00800 Colombo, Sri Lanka

## Abstract

*Introduction*. Cadaveric dissections and prosections have traditionally been part of undergraduate medical teaching.* Materials and Methods*. Hundred and fifty-nine first-year students in the Faculty of Medicine, University of Colombo, were invited to participate in the above study. Students were randomly allocated to two age and gender matched groups. Both groups were exposed to identical series of lectures regarding anatomy of the abdomen and conventional cadaveric prosections of the abdomen. The test group (*n* = 77, 48.4%) was also exposed to cadaveric cross-sectional slices of the abdomen to which the control group (*n* = 82, 51.6%) was blinded. At the end of the teaching session both groups were assessed by using their performance in a timed multiple choice question paper as well as ability to identify structures in abdominal CT films.* Results*. Scores for spatial and radiological anatomy were significantly higher among the test group when compared with the control group (*P* < 0.05, CI 95%). Majority of the students in both control and test groups agreed that cadaveric cross section may be useful for them to understand spatial and radiological anatomy.* Conclusion*. Introduction of cadaveric cross-sectional prosections may help students to understand spatial and radiological anatomy better.

## 1. Introduction

Cadaveric prosections have been fundamental to understanding the complexities of the human body structure and have played a pivotal role in gross anatomy teaching in medical education [1]. Dissection of cadavers provides student with the unique perception of intricacies of human anatomy that is thought to facilitate understanding of complex visuospatial relationships of the human body [2]. Unfortunately the scarcity of cadavers, adverse student cadaver ratio, and increasingly shorter courses have made cadaveric dissection redundant from anatomy curricula [3]. An increasing number of computer assisted learning tools (CAL) have been introduced to fill in the practical aspects of teaching human anatomy [4–6]. In the absence of cadaveric dissection, prosected specimens have increasingly been used in teaching anatomy to medical undergraduates [7]. Newer techniques like plastination have been developed and used increasingly. With the advent of sophisticated imaging modalities such as 3D CT reconstructions, high resolution cadaveric CT scans have found its way to anatomy laboratories [8]. Introduction of ultrasound in anatomy teaching session has been received with much enthusiasm [9].

Traditionally anatomy has been taught on a regional basis. However cross-sectional anatomy is routinely encountered by clinicians. With the advent of modern medical imaging, more and more cross-sectional images of human anatomy are available as teaching material for modern day students. Can we improve spatial understanding of anatomy and imaging by exposing students to cadaveric cross-sectional specimens? We attempted to answer this question in a carefully designed case control study.

## 2. Materials and Methods

Hundred and fifty-nine (male *n* = 84, 52.8% female *n* = 75, 47.2%) first-year students in the Faculty of Medicine, University of Colombo, were invited to participate in the above study. Written informed consent was obtained and the consenting students were randomly allocated to two age and gender matched groups. Both groups were exposed to identical series of lectures regarding anatomy of the abdominal cavity. Control group was exposed to conventional cadaveric prosections of the abdomen. The test group (*n* = 77, 48.4%) was exposed to a session containing prosected cadaveric cross-sectional slices of the abdomen to which the control group (*n* = 82, 51.6%) was blinded. At the end of the teaching session understanding of the spatial anatomy of the abdomen among both groups was assessed by using their performance in a timed pretested and validated multiple choice question paper (SA). In addition ability to identify structures in abdominal CT films was also tested in a structured examination using abdominal CT scan films (RA). Individual marks of each component were added to arrive at the final cumulative scores (CS).

## 3. Ethical Considerations

Written informed consent was obtained from all participants. Ethics clearance was obtained from University of Colombo Ethics Committee.

## 4. Results

### 4.1. Demography (Table 1)

159 (male *n* = 84, 52.8% female *n* = 75, 47.2%) first-year medical students participated in the study. Male female distribution and mean age were nearly equal in both groups.

### 4.2. Scores for Spatial and Radiological Anatomy (Table 2)

Scores for spatial anatomy (SA) and radiological anatomy (RA) as well as the cumulative scores (CS) were higher among the test group when compared with the control group. Differences observed among the scores were statistically significant (*P* < 0.05, CI 95%).

Subgroup analysis among control and test groups failed to reveal any statistically significant difference in scores between male and female students.

### 4.3. Perceptions (Table 3)

After the quiz was completed, control group was also exposed to a brief session of prosected cross-sectional specimens similar to the test group, and both groups were requested to complete the perceptions section. Majority of the students in both control and test groups agreed that cadaveric cross section may be useful for them to understand spatial and radiological anatomy (97.6% and 98.7%, resp.). Similarly vast majority in both groups (control 97.6% test 97.4%) also thought that radiological imaging may be used more often as adjunct to teaching anatomy.

## 5. Discussion

Sound knowledge of anatomy has been considered cornerstone of medical education [10]. This is especially true in surgical education where clear understanding of spatial relationship of structures with one another is extremely important. Even though increasingly less time is devoted to teaching anatomy in conventional curricula, clinicians are exposed to more and more cross-sectional anatomy with the sophistication of the medical imaging [11].

Certainly the concept of using cross-sectional imaging as an adjunct for teaching anatomy is not new. As early as 1985, 68% of medical schools in the United States were using some form of radiological imaging in undergraduate teaching [12]. Over time the imaging modalities used for teaching has changed with the changing trends of practice of medicine-predominantly plain X-rays and fluoroscopy [13] evolving onto cross-sectional imaging such as CT and MRI [11]. Some medical schools have taken this approach to the extreme with completely doing away with cadaveric dissection/prosections from the curricula, exclusively relying on medical imaging [14], although many would argue against such a radical shift from the established teaching practices [15].

Early ingenious attempts have been made to incorporate cross-sectional anatomy into medical curricula. Oh and colleagues used clay models of internal organs and used cut surfaces of the models to teach students how to interpret cross-sectional diagnostic imaging [16]. Plastic models have been used in the early days to circumvent problems associated with cadaveric dissection [14]. Interestingly body painting has been used to promote understanding of surface anatomy and its relation with underlying structures [17]. With the advancement of plastination an effective technique for tissue preservation, more and more plastinated cross sections were introduced onto cadaveric anatomical courses [18]. With further advancement of technology, computer assisted learning (CAL) was introduced. Term anatomical informatics was introduced in 2002 by Trelease. Various web based applications and computer based software have been developed to aid the student to grasp key anatomical concepts. Indeed it has been shown that students who had access to computer based anatomical programs scored consistently higher in professional examinations [19]. Utilization of modern technology in teaching anatomy has been uplifted to a new level by the advent of visible human anatomy projects [5, 6].

In our study we noted statistically significant improvement in spatial anatomical scores as well as radiological anatomy interpretation scores when students were exposed to cadaveric cross sections. Although there are multiple reviews on use of cross-sectional radiological imaging material as teaching aids for students [12–14], hardly any exist which objectively look at the utility of cadaveric cross sections, in improving students spatial understanding of anatomical structures. Indeed modern medical imaging relies heavily on cross-sectional anatomy, and it is logical to incorporate cadaveric cross sections into preclinical anatomy curricula. Due to this fact, test group students who were exposed to cross-sectional prosections scored significantly higher marks in interpretation of radiological imaging also.

Our study also explored the perceptions of the students regarding use of cadaveric cross-sectional imaging in anatomy teaching. Interestingly enough, significant proportion of both test and control groups thought that cross-sectional prosections may be extremely helpful in understanding anatomical concepts. Even though we did not use imaging as a teaching tool in our study, majority of both test and control groups felt that radiological imaging would be a useful adjunct to learning anatomy. This is in keeping with many other studies where integration of radiological anatomy to traditional anatomy courses was received with welcome arms by both students and instructors [9].

Although we propose to use cadaveric prosections and radiological imaging as an adjunct to conventional anatomy teaching we still do not advocate sole reliance of cross-sectional imaging for purposes of teaching anatomy. Indeed there have been rising concerns regarding falling quality of anatomical education with ever decreasing course times and limited availability of cadavers for dissection [15]. Although hybrid teaching with imaging material will undoubtedly aid conceptual understanding and retention of facts, it should not be considered a substitute for conventional cadaver based anatomy courses [20].

## 6. Conclusion

Our study illustrates that cadaveric cross-sectional prosections can be used as an adjunct to improve spatial anatomical knowledge among students and to improve the ability to accurately interpret the radiological imaging. We recommend incorporation of prosected cadaveric cross sections as well as radiological imaging material as adjuncts to conventional anatomical curricula.

## Figures and Tables

**Table 1:** Basic demographic data for the test and control.

	Male	Female	Mean age (years)
Control group	40 (48.8)	42 (51.2)	21.3
Test group	44 (57.1)	33 (42.9)	20.9

	84 (52.8%)	75 (47.2%)	

**Table 2:** Comparison of scores between test and control groups value *P* < 0.05, CI 95%.

	Control group	Test group	*P* value
RA	25.85 [SD = 11.05]	42.01 [SD = 3.99]	<0.05
SA	33.40 [SD = 7.35]	37.59 [SD = 4.43]	<0.05
CS	58.89 [SD = 13.55]	79.29 [SD = 6.54]	<0.05

RA: radiological anatomy, SA: spatial anatomy, and CS: combined scores.

**Table 3:** Subgroup analysis of the mean scores of control and test groups.

	Male	Female	*P* value
Control			
RA	28.30 (SD 11.50)	23.52 (SD 10.21)	0.0502
SA	33.40 (SD 6.98)	33.40 (SD 7.78)	0.9952
CS	61.70 (SD 14.93)	56.92 (SD 11.95)	0.1172

Test			
RA	42.04 (SD 3.78)	41.97 (SD 4.32)	0.941
SA	37.18 (SD 4.82)	38.15 (SD 3.85)	0.3318
CS	79.22 (SD 6.58)	79.39 (SD 6.60)	0.9164

## Abbreviations

CT: Computed Tomography
MRI: Magnetic Resonance Imaging
SA: Spatial anatomy
RA: Radiological anatomy
CS: Cumulative scores
CAL: Computer assisted learning.

## References

[1] McLachlan J. C., Patten D. (2006). Anatomy teaching: ghosts of the past, present and future. *Medical Education*.

[2] McLachlan J. C., Bligh J., Bradley P., Searle J. (2004). Teaching anatomy without cadavers. *Medical Education*.

[3] Gregory J. K., Lachman N., Camp C. L., Chen L. P., Pawlina W. (2009). Restructuring a basic science course for core competencies: an example from anatomy teaching. *Medical Teacher*.

[4] Temkin B., Acosta E., Malvankar A., Vaidyanath S. (2006). An interactive three-dimensional virtual body structures system for anatomical training over the internet. *Clinical Anatomy*.

[5] Park J. S., Chung M. S., Hwang S. B., Shin B.-S., Park H. S. (2006). Visible Korean human: its techniques and applications. *Clinical Anatomy*.

[6] Zhang S.-X., Heng P.-A., Liu Z.-J. (2006). Chinese visible human project. *Clinical Anatomy*.

[7] Collins J. P. (2008). Modern approaches to teaching and learning anatomy. *British Medical Journal*.

[8] Durosaro O., Lachman N., Pawlina W. (2008). Use of knowledge-sharing web-based portal in gross and microscopic anatomy. *Annals of the Academy of Medicine Singapore*.

[9] Rao S., Van Holsbeeck L., Musial J. L. (2008). A pilot study of comprehensive ultrasound education at the Wayne State University School of Medicine: a pioneer year review. *Journal of Ultrasound in Medicine*.

[10] Bergman E. M., Van Der Vleuten C. P. M., Scherpbier A. J. J. A. (2011). Why don't they know enough about anatomy? A narrative review. *Medical Teacher*.

[11] Ganske I., Su T., Loukas M., Shaffer K. (2006). Teaching methods in anatomy courses in North American medical schools. The role of radiology. *Academic Radiology*.

[12] Bassett L. W., Squire L. F. (1985). Anatomy instruction by radiologists. *Investigative Radiology*.

[13] Johnson T. H. (1969). Medical school radiology teaching and examination methods. *Radiology*.

[14] McLachlan J. C. (2004). New path for teaching anatomy: living anatomy and medical imaging vs. dissection. *Anatomical Record—Part B: New Anatomist*.

[15] Ellis H. (2001). Teaching in the dissecting room. *Clinical Anatomy*.

[16] Oh C.-S., Kim J.-Y., Choe Y. H. (2009). Learning of cross-sectional anatomy using clay models. *Anatomical Sciences Education*.

[17] Philip C. T., Unruh K. P., Lachman N., Pawlina W. (2008). An explorative learning approach to teaching clinical anatomy using student generated content. *Anatomical Sciences Education*.

[18] Dhingra R., Taranikanti V., Kumar R. (2006). Plastination: teaching aids in anatomy revisited. *National Medical Journal of India*.

[19] McNulty J. A., Sonntag B., Sinacore J. M. (2009). Evaluation of computer-aided instruction in a gross anatomy course: a six-year study. *Anatomical Sciences Education*.

[20] Gunderman R. B., Wilson P. K. (2005). Viewpoint: exploring the human interior: the roles of cadaver dissection and radiologic imaging in teaching anatomy. *Academic Medicine*.

